# Observational study on stability of within-day glycemic variability of type 2 diabetes inpatients treated with decoctions of traditional Chinese medicine

**DOI:** 10.3389/fphar.2024.1378140

**Published:** 2024-07-19

**Authors:** Ying Xing, Penghui Li, Guoming Pang, Hui Zhao, Tiancai Wen

**Affiliations:** ^1^ Institute of Information on Traditional Chinese Medicine, China Academy of Chinese Medical Sciences, Beijing, China; ^2^ Traditional Chinese Medicine Data Center, China Academy of Chinese Medical Sciences, Beijing, China; ^3^ Kaifeng Traditional Chinese Medicine Hospital, Henan, China; ^4^ China Center for Evidence-Based Traditional Chinese Medicine, China Academy of Chinese Medical Sciences, Beijing, China

**Keywords:** within-day glycemic variability, glycemic fluctuations, glycemic stability, type 2 diabetes, traditional Chinese medicine decoction therapy

## Abstract

**Background:**

Within-day glycemic variability (GV), characterized by frequent and significant fluctuations in blood glucose levels, is a growing concern in hospitalized patients with type 2 diabetes mellitus (T2DM). It is associated with an increased risk of hypoglycemia and potentially higher long-term mortality rates. Robust clinical evidence is needed to determine whether traditional Chinese medicine (TCM) decoctions can be a beneficial addition to the management of within-day GV in this patient population.

**Methods:**

This retrospective cohort study utilized data from adult inpatients diagnosed with T2DM admitted to the Traditional Chinese Medicine Hospital of Kaifeng. The primary outcome investigated was the association between the use of TCM decoctions and improved stability of within-day GV. Blood glucose variability was assessed using the standard deviation of blood glucose values (SDBG). For each patient, the total number of hospitalization days with SDBG below 2 mmol/L was calculated to represent within-day GV stability. Hospitalization duration served as the secondary outcome, compared between patients receiving TCM decoctions and those who did not. The primary analysis employed a multivariable logistic regression model, with propensity score matching to account for potential confounding variables.

**Results:**

A total of 1,360 patients were included in the final analysis. The use of TCM decoctions was significantly associated with enhanced stability of within-day GV (OR = 1.77, 95% CI: 1.34–2.33, *P* < 0.01). This association was most prominent in patients with a diagnosis of deficiency syndrome (predominantly qi-yin deficiency, accounting for 74.8% of cases) and a disease duration of less than 5 years (OR = 2.28, 95% CI: 1.21–4.29, *P* = 0.03). However, TCM decoctions did not exert a statistically significant effect on hospitalization duration among patients with T2DM (OR = 0.96, 95% CI: 0.91–1.01, *P* = 0.22).

**Conclusion:**

This study suggests that TCM decoctions may be effective in improving within-day GV stability in hospitalized patients with T2DM. This effect appears to be most pronounced in patients diagnosed with deficiency syndrome, particularly those with qi-yin deficiency and a shorter disease course. Further investigation is warranted to confirm these findings and elucidate the underlying mechanisms.

## Introduction

While glycated hemoglobin (HbA1c) has served as the gold standard for assessing glycemic control in patients with type 2 diabetes, its sole reliance for this purpose may be inadequate (Hirsch, 2015). This limitation stems from the inherent nature of HbA1c, which reflects average blood glucose levels over the preceding 2–3 months, failing to capture daily acute fluctuations or hypoglycemic episodes. Consequently, patients with T2D can exhibit significant glycemic variability (GV) even when achieving target HbA1c levels (Kovatchev and Cobelli, 2016; Dandona, 2017). GV, characterized by short-term oscillations in plasma glucose, typically refers to fluctuations within a 24-h window, known as within-day GV (Julla et al., 2021). Frequent and extensive within-day GV independently increases the risk of T2D inpatients experiencing hypoglycemia (Kauffmann et al., 2011; Monnier et al., 2011; Bajaj et al., 2017) and cardiovascular complications (Monnier et al., 2006; Saisho, 2014; Nusca et al., 2018; Scott et al., 2020). This association has further been linked to prolonged hospital stays and increased long-term mortality rates (Mendez et al., 2013; Akirov et al., 2017; Timmons et al., 2017; Akirov et al., 2019; Jordán-Domingo et al., 2021). The growing recognition of stable within-day GV’s importance in T2D management highlights its potential as a novel target for glycemic control therapy.

Traditional Chinese medicinal (TCM) decoction therapy is widely used in diabetes management due to its perceived gentle effects and reported glucose-lowering efficacy (Hu and Jia, 2019). However, robust clinical evidence regarding the effectiveness of TCM decoctions in specifically reducing within-day glycemic variability (GV) and promoting glycemic stability remains limited. This study aimed to investigate the association between the administration of oral TCM decoctions and the maintenance of stable within-day GV in hospitalized patients diagnosed with type 2 diabetes mellitus (T2DM). The primary objective was to compare the impact of TCM decoctions on within-day GV stability with that of conventional antidiabetic Western medications in this patient population. Besides, we sought to evaluate the potential benefits of TCM decoctions in reducing the duration of hospitalization.

## Methods

### Data sources and participants

This retrospective cohort study utilized the electronic medical record (EMR) database of the Department of Endocrinology at Kaifeng Traditional Chinese Medicine Hospital in Henan Province, China. The database encompassed demographic information, vital signs, past and current diagnoses, clinical symptoms, laboratory results, medication administration details (including both traditional Chinese medicine and Western medications), and self-monitoring of blood glucose (SMBG) data for 12,664 patients admitted between 1 January, 2017, and 16 June 2021. The datasets were linked using unique patient identifiers.

**FIGURE 1:**
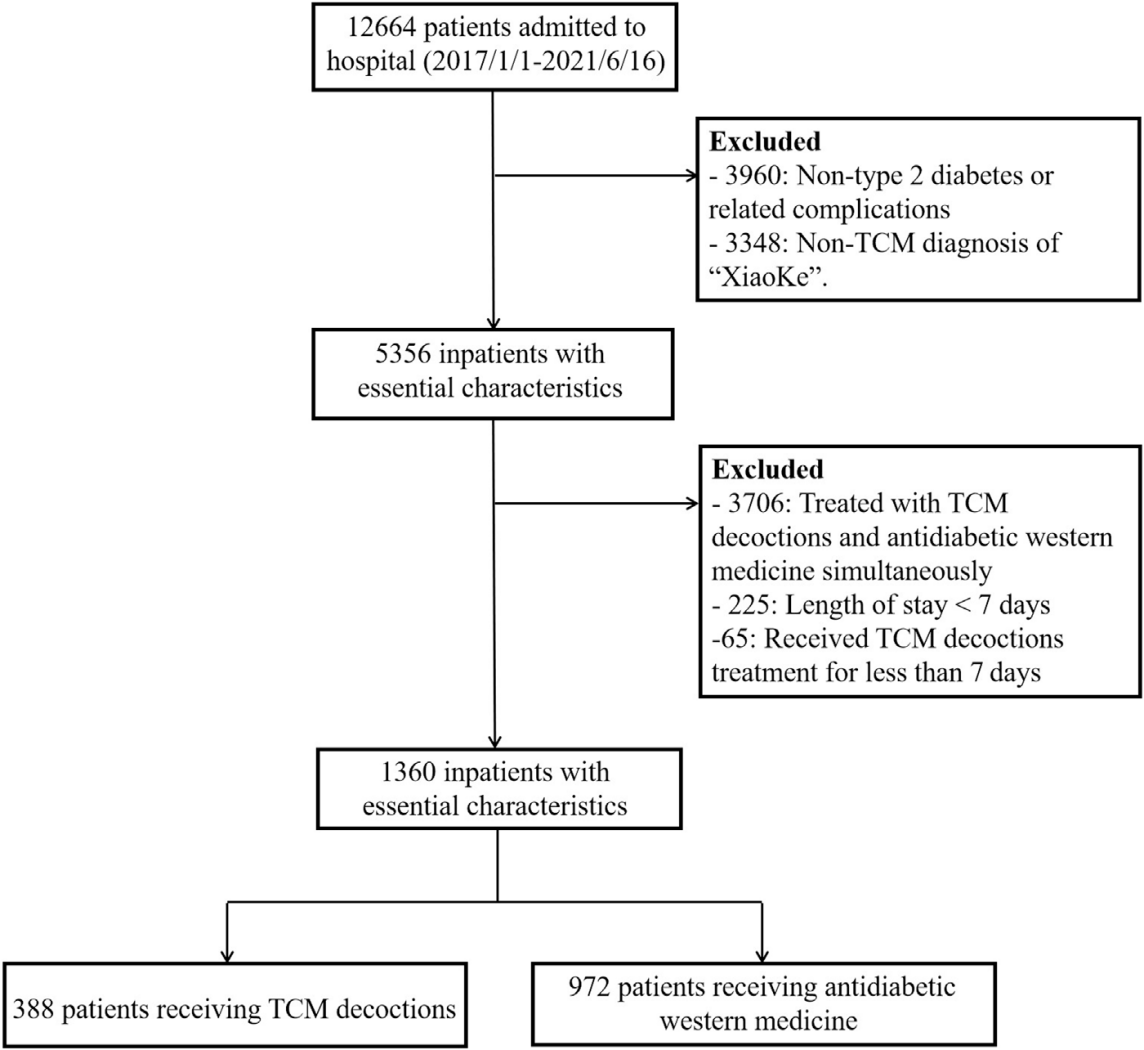
Flowchart of study population selection.

The study cohort comprised hospitalized adult patients (aged ≥ 18 years) diagnosed with type 2 diabetes mellitus. Patients were categorized into two groups: those receiving TCM decoctions (TCM treatment group) and those receiving antidiabetic Western medications (non-TCM treatment group). Medical records with admission diagnoses other than T2DM or its complications were excluded. Additionally, patients whose TCM diagnosis did not correspond to the term “XiaoKe” (representing T2DM in TCM theory) were excluded. To minimize the influence of repeated hospitalizations, patients with multiple admissions within the study timeframe were excluded if the interval between admissions was less than 1 year. Admissions exceeding 1 year from the previous admission were considered new admissions. Patients receiving both TCM decoctions and antidiabetic Western medications concurrently were also excluded. To ensure adequate exposure to TCM decoction treatment, patients with a hospitalization duration of less than 7 days and receiving TCM decoctions fewer than seven times were excluded. The study baseline was defined as 24 h after patient admission. All laboratory tests were performed on fasting blood samples collected within 24 h of admission. The second-day SMBG data served as the entry point for patients into the cohort. The final analysis included 1,361 patients (Figure 1).

This study was conducted in accordance with ethical principles governing real-world research and non-interventional studies. All data pertaining to research participants received approval from the Ethics Committee of Kaifeng Traditional Chinese Medicine Hospital approval number: 2022-ky-006.

### Covariates

In our analysis, we adjusted for potential confounding variables including age, gender, duration of diabetes, body mass index (BMI), baseline HbA1c, hypertension, LDL cholesterol, fasting insulin levels, C-peptide, average fasting plasma glucose (FPG) during hospitalization, comorbidities, and TCM syndrome. Diabetes duration was categorized into four groups: 0 to less than 3 years, 3 to less than 5 years, 5 to less than 10 years, and 10 or more years. Following the consensus of Chinese experts in medical nutrition therapy for overweight/obesity (CecCooOMN, 2016), BMI was divided into four categories: less than or equal to 18 kg/m^2^, 18–23.9 kg/m^2^, 24–27.9 kg/m^2^, and greater than or equal to 28 kg/m^2^. LDL cholesterol, fasting insulin level, and C-peptide were also categorized. LDL was classified as either less than 2.6 mmol/L or greater than or equal to 2.6 mmol/L. Fasting insulin levels were grouped as less than 10 μU/mL, 10–15 μU/mL, and greater than 15 μU/mL. C-peptide levels were categorized as less than 1.71 ng/mL, 1.71–2.51 ng/mL, and greater than or equal to 2.51 ng/mL. We considered 13 common comorbidities associated with diabetes, including liver diseases, hypertension, hyperlipidemia, osteoarthropathy, coronary atherosclerosis, chronic kidney disease, cerebral infarction, ischemic cerebrovascular disease, ischemic heart disease, diabetic retinopathy, diabetic macrovascular disease, diabetic polyneuropathy, and diabetic peripheral vascular disease. Finally, TCM syndromes were categorized into four groups: deficiency syndromes, phlegm syndromes, liver stagnation and spleen deficiency syndrome, and dampness-heat syndrome.

### Exposures

All patients in the TCM treatment group received decoctions according to the TCM treatment modalities for Type 2 diabetes as practiced at Kaifeng Traditional Chinese Medicine Hospital (Pang et al., 2019). The primary decoctions administered included: 1) Qingre Yangyin Tiaotang decoction; 2) Yiqi Yangyin Tiaotang Decoction; 3) Shugan Jianpi Tiaotang decoction; 4) Hezhong Jiangzhuo tiaotang decoction; 5) Qingre Huashi Tiaotang decoction; and 6) Jianpi Yishen Tiaotang decoction (details of the composition of each decoction are provided in Supplementary Table S1). Additional classical TCM decoctions, such as Shenqi Dihuang Tang, were also considered (complete description of classical TCM decoctions summarized in Supplementary Table S1).

The selection and adjustment of these decoctions were carried out by experienced TCM practitioners, adhering to the principles of TCM syndrome differentiation, tailored to the individual symptoms of each patient. Each participant received 400 mL of the decoction daily, divided into two doses of 200 mL each, taken in the morning and evening. The duration of treatment was no less than seven consecutive days. Patients assigned to the TCM group were permitted to receive usual care medications; however, the concurrent use of any Western medications or Commercial Chinese Polyherbal Preparation with known glucose-lowering effects was strictly prohibited.

The non-TCM treatment group received standard Western medical treatment for hyperglycemia, as outlined in the Chinese guidelines for the prevention and treatment of type 2 diabetes (Jia et al., 2019). These therapies included oral anti-hyperglycemic agents (Thiazolidinediones, Glucagon-like peptide1 agonist, Dipeptidyl peptidase-4 inhibitors, Glinide, SGLT2 inhibitors, Sulfonylurea, α-glucosidase inhibitors, and Metformin) and insulin, with the specific medication regimen selected by attending physicians based on each patient’s individual clinical presentation and adherence to the routine diabetes management protocol for inpatients at Kaifeng Traditional Chinese Medicine Hospital. Treatment selection for the control group was not prescriptive, allowing for flexibility based on individual needs.

### Outcomes

The primary outcome of interest was the stability of within-day GV. This was assessed through the standard deviation of blood glucose values (SDBG) measured by SMBG seven times a day (fasting, pre-breakfast, pre-lunch, post-lunch, pre-dinner, post-dinner, and bedtime). Following the expert consensus on diabetes mellitus glycemic variability management (CSo, 2017), a threshold of SDBG measured by SMBG less than 2 mmol/L was considered indicative of normal within-day GV for Chinese patients with diabetes. To quantify the stability of within-day GV, we calculated a score for each patient. This score reflected the proportion of hospitalization days with SDBG below 2 mmol/L. We achieved this by dividing the total number of days with SDBG below 2 mmol/L by the total number of hospitalization days and multiplying by 100. For example, consider a patient with a sequence of SDBG values: 6, 5, 0.8, 2.3, 2.6, 1.5, 1.6, 0.9, 3.6, 2.3, 2.2, and 2.7 mmol/L. If the total length of stay was 12 days and the number of days with SDBG below 2 mmol/L was four, the score would be 33 [i.e., (100 × 4)/12]. For analysis, scores were categorized into four groups: 0–20, 21–40, 41–60, and ≥ 61. For sensitivity analyses, we considered the postprandial glucose excursion (PPGE) and largest amplitude of glycemic excursions (LAGE) as additional assessment indicators for within-day GV stability (CSo, 2017). Similarly, we calculated the total number of days within the expected range for PPGE and LAGE as a measure of within-day GV stability. The secondary outcome was length of hospital stay.

### Statistical analysis

Associations between treatment modality and patient demographic, clinical, and other baseline characteristics were assessed using a t-test for continuous variables and chi-square tests for categorical variables.

To investigate the association between the use of TCM decoctions and the stability of within-day GV, we employed a multivariable logistic regression model. Similarly, a Poisson regression model was used to assess the relationship between hospital stay duration and TCM decoction use. Our initial multivariable logistic regression and Poisson regression models included all potential covariates identified for the study. However, to account for potential confounding variables due to the non-randomized nature of TCM decoction administration, we implemented propensity score matching techniques. Prior to propensity score matching, we conducted a univariate analysis of all potentially explanatory factors for both the primary (within-day GV stability) and secondary (hospital stay duration) outcomes. This analysis identified 15 factors significantly associated with the primary outcome and 7 factors associated with the secondary outcome (all *p*-values < 0.05, detailed in Supplementary Table S2). We then utilized a logistic regression model to estimate a propensity score for each patient. This score represented the predicted probability of receiving TCM decoctions, and it was based on the 15 significant factors identified for the primary outcome (7 factors for the secondary outcome). Finally, we employed three propensity score matching methods within separate multivariable logistic regression and Poisson regression models to estimate the association between TCM decoction use and within-day GV stability and hospital stay duration, respectively.

The primary analysis employed propensity score matching to address potential confounding due to the non-randomized treatment assignment. Patients receiving TCM decoctions were matched in a 1:1 ratio to those receiving antidiabetic drugs based on their predicted probabilities of receiving TCM decoctions derived from the propensity score model. A greedy nearest neighbor matching algorithm was used with a maximum caliper width of ±1%, ensuring a close match between patients in the TCM and non-TCM groups on all measured covariates. Logistic regression and Poisson regression models were then performed using the propensity score-matched cohort data to estimate the association between TCM decoction use and the primary and secondary outcomes, respectively.

To further explore the robustness of our findings, we performed two additional prespecified sensitivity analyses for the primary outcome, investigating within-day glycemic variability stability. The first analysis employed inverse probability of treatment weighting (IPTW) on the propensity score. The second analysis incorporated the propensity score itself as an additional covariate in the regression model. Furthermore, we conducted sensitivity analyses to assess the potential influence of missing data. Here, we defined within-day GV stability using the standard deviation of %CV_w_ values during hospitalization and utilized a complete data set for analysis.

We conducted prespecified subgroup analyses to explore the impact of TCM decoctions on within-day glycemic variability stability within specific patient populations. Patients in the TCM and non-TCM treatment groups were matched based on three criteria: TCM syndrome (deficiency syndrome vs. phlegm syndromes/liver stagnation and spleen deficiency syndrome), diabetes duration (≤5 years vs. >5 years), and the logit of the propensity score. Within each of the four resulting subgroups, a multivariable logistic regression model was used to compare the risk reduction for the primary outcome (reduced within-day GV instability) between the TCM and non-TCM treatment groups.

To address missing covariate data and minimize selection bias, we employed multivariate imputation by chained equations. This approach estimates missing values under the assumption that data are missing at random. Prior to imputation, a missing pattern analysis confirmed the presence of only monotonic missing patterns (i.e., missing data only occurred for subsequent variables in a sequence). Five covariates (BMI, LDL cholesterol, fasting insulin, C-peptide, and duration of diabetes) contained missing data, ranging from 0% to 14% across variables. Notably, 65% of patients had complete data for all covariates. To account for imputation uncertainty, five separate imputed datasets were generated for analysis. The results from each imputed dataset were subsequently pooled to estimate regression parameters.

Statistical analyses were performed using R software version 4.3.1. A two-sided *p*-value less than 0.05 was considered statistically significant. For the propensity score matched cohort, a standardized difference of less than 0.1 between the TCM decoction and control groups indicated a good balance on all types of distributional characteristics between the two groups.

## Results

### Characteristics of the cohort

A total of 1,360 patients were included in the final analysis (Figure 1). The distribution of patients across the within-day glycemic variability (GV) stability score categories was as follows: 0–20 (n = 474), 21–40 (n = 398), 41–60 (n = 267), and ≥ 61 (n = 221).

Table 1 summarizes the baseline characteristics of the study participants before propensity score matching, categorized by exposure to TCM decoctions. Among the 1,360 patients, 388 (28.5%) received TCM decoctions, while the remaining 972 (71.5%) did not. In the unmatched sample, statistically significant differences in exposure to TCM decoctions were observed based on age, sex, BMI, duration of diabetes, HbA1c, FPG, fasting insulin levels, C-peptide levels, TCM syndrome diagnosis, and the presence of several comorbidities. Notably, 53.1% of patients treated with TCM decoctions had a diabetes duration of less than 3 years, while over 60% of patients who did not receive TCM decoctions had a duration of 5–10 years or longer. Additionally, patients receiving TCM decoctions exhibited lower baseline HbA1c levels compared to those who did not [median: 7.5% (9.4 mmol/L) vs. 9.5% (12.6 mmol/L)].

**TABLE 1:** Participant characteristics before propensity-score matching^a^.

Characteristic	Non-TCM decoctions (N = 972)	TCM decoctions (N = 388)	*P* value
Age -- mean (SD), year	57.3 (12.7)	53.1 (13.3)	<0.01
Sex -- no. (%)			
Female	555 (57.1)	269 (69.3)	<0.01
Male	417 (42.9)	119 (30.7)	
Body-mass index -- no. (%), kg/m^2^			
≤18 and 18–23.9	290 (32.2)	87 (24.3)	<0.01
24–27.9	422 (46.8)	176 (49.2)	
≥28	190 (21.1)	95 (26.5)	
Duration of diabetes -- no. (%), year			
<3	287 (29.6)	206 (53.1)	<0.01
3–5	95 (9.8)	52 (13.4)	
5–10	202 (20.8)	79 (20.4)	
≥10	387 (39.9)	51 (13.1)	
Length of stay -- median (IQR), day	13.0 (11.0, 15.0)	12.0 (9.0, 15.0)	<0.01
Systolic blood pressure -- median (IQR), mm HG	130.0 (120.0–140.0)	130.0 (120.0–140.0)	0.19
HbA1c -- median (IQR), %	9.5 [12.6 mmol/L] [8.1 (10.3 mmol/L)−11.1 (15.1 mmol/L)]	7.5 [9.4 mmol/L] [6.6 (7.9 mmol/L)−8.7 (11.3 mmol/L)]	<0.01
FPG -- median (IQR), mmol/L	8.8 (8.2–10.6)	8.3 (7.9–8.7)	<0.01
Fasting insulin -- no. (%), μIU/mL			
<10	466 (57.7)	164 (45.8)	<0.01
10–15	203 (25.1)	100 (27.9)	
>15	139 (17.2)	94 (26.3)	
LDL -- no. (%), mmol/L			
<2.6	312 (36.7)	137 (41.3)	0.18
≥2.6	538 (63.3)	195 (58.7)	
C-peptide -- no. (%), ng/mL			
<1.17	115 (14.2)	15 (4.2)	<0.01
1.17–2.51	231 (28.6)	76 (21.2)	
≥2.51	462 (57.2)	267 (74.6)	
TCM syndrome -- no. (%)			
Deficiency syndromes	559 (57.5)	167 (43.0)	<0.01
Phlegm syndromes	219 (22.5)	117 (30.2)	
Liver stagnation and spleen deficiency syndrome	107 (11.0)	54 (13.9)	
Dampness-heat syndrome	87 (9.0)	50 (12.9)	
Comorbidities -- no. (%)			
Liver diseases	192 (19.8)	97 (25.0)	0.04
Hypertension	396 (40.7)	156 (40.2)	0.88
Hyperlipidemia	134 (13.8)	79 (20.4)	<0.01
Osteoarthropathia	70 (7.2)	34 (8.8)	0.39
Coronary atherosclerosis	206 (21.2)	73 (18.8)	0.35
Chronic kidney disease	315 (32.4)	85 (21.9)	<0.01
Cerebral infarction	123 (12.7)	44 (11.3)	0.56
Ischemic cerebrovascular disease	111 (11.4)	33 (8.5)	0.14
Ischemic heart disease	85 (8.7)	20 (5.2)	0.03
Diabetic retinopathy	192 (19.8)	25 (6.4)	<0.01
Diabetic macrovascular disease	96 (9.9)	22 (5.7)	0.02
Diabetic polyneuropathy	491 (50.5)	127 (32.7)	<0.01
Diabetic peripheral vascular disease	100 (10.3)	30 (7.7)	0.17

^a^ In the unmatched analysis, data on the BMI level were missing for 100 patients, on the LDL level for 178, on the Fasting insulin level for 194, on the c-peptide level for 194, on the duration of diabetes level for 1.

Following propensity score matching, a total of 282 matched patient pairs were obtained (Table 2). Importantly, there were no significant differences (*p* > 0.30 for all comparisons) between the TCM decoction and non-TCM decoction groups across any of the 17 baseline characteristics. Additionally, the standardized mean differences between the two groups were all less than 0.1, indicating successful covariate balancing through propensity score matching.

**TABLE 2:** Participant characteristics after propensity-score matching.^a^

Characteristic	TCM decoctions (N = 278)	Non-TCM decoctions (N = 278)
Age -- mean (SD), year	54.1 (12.3)	54.1 (14.0)
Body-mass index -- no. (%), kg/m^2^		
≤18 and 18–23.9	69 (24.8)	66 (23.7)
24–27.9	136 (48.9)	136 (48.9)
≥28	73 (26.3)	76 (27.3)
Duration of diabetes -- no. (%), year		
<3	121 (43.5)	127 (45.7)
3–5	43 (15.5)	40 (14.4)
5–10	58 (20.9)	64 (23.0)
≥10	56 (20.1)	47 (16.9)
Systolic blood pressure -- median (IQR), mm HG	130.0 (120.0–140.0)	130.0 (120.0–140.0)
HbA1c -- median (IQR), %	7.8 [9.8 mmol/L] [7.0 (8.6 mmol/L)−8.9 (11.6 mmol/L)]	8.0 [10.2 mmol/L] [7.0 (8.6 mmol/L)−9.0 (11.8 mmol/L)]
FPG -- median (IQR), mmol/L	8.30 (7.9–9.1)	8.30 (8.0–9.0)
Fasting insulin -- no. (%), μIU/mL		
<10	134 (48.2)	128 (46.0)
10–15	82 (29.5)	81 (29.1)
>15	62 (22.3)	69 (24.8)
C-peptide -- no. (%), ng/mL		
<1.17	14 (5.0)	14 (5.0)
1.17–2.51	69 (24.8)	58 (20.9)
≥2.51	195 (70.1)	206 (74.1)
TCM syndrome -- no. (%)		
Deficiency syndromes	130 (46.8)	118 (42.4)
Phlegm syndromes	81 (29.1)	81 (29.1)
Liver stagnation and spleen deficiency syndrome	34 (12.2)	40 (14.4)
Dampness-heat syndrome	33 (11.9)	39 (14.0)
Comorbidities -- no. (%)		
Liver diseases	58 (20.9)	67 (24.1)
Hyperlipidemia	57 (20.5)	57 (20.5)
Coronary atherosclerosis	56 (20.1)	54 (19.4)
Chronic kidney disease	66 (23.7)	69 (24.8)
Cerebral infarction	31 (11.2)	36 (12.9)
Diabetic retinopathy	28 (10.1)	23 (8.3)

^a^ Multiple imputation was used to account for missing data in the propensity-score-matched analysis.

### Primary endpoint

Overall, patients who received TCM decoctions exhibited greater stability of within-day glycemic variability compared to those who did not. This association was statistically significant in both unadjusted and adjusted analyses. In the unadjusted analysis, the odds ratio (OR) for improved within-day GV stability with TCM decoction use was 3.15 [95% confidence interval (CI): 2.62 to 3.78; *p* < 0.01; Table 3]. This finding remained significant after adjusting for potential confounding variables using propensity score matching (OR: 1.77; 95% CI: 1.34 to 2.33; *p* < 0.01; Table 3). Similar statistically significant results were obtained in both the adjusted analysis (OR: 1.77; 95% CI: 1.58 to 1.98; *p* < 0.001; Table 3) and the inverse probability weighted analysis (OR: 1.63; 95% CI: 1.32 to 2.04; *p* < 0.001; Table 3) based on propensity score. The observed association between TCM decoctions and improved within-day GV stability remained consistent when using postprandial glucose excursion (PPGE) and largest amplitude of glycemic excursions (LAGE) as alternative assessment indicators. The results of the sensitivity analysis are in Supplementary Table S3. Additionally, the analysis of complete cases excluding missing data yielded comparable results (Supplementary Table S3). Subgroup analyses by TCM syndrome and diabetes duration categories revealed a significant benefit for patients diagnosed with deficiency syndromes (predominantly deficiency of qi-yin, accounting for 74.8%) and a diabetes duration of less than 5 years (OR: 2.28; 95% CI: 1.21 to 4.29; *p* = 0.03; Table 4). However, no statistically significant benefits were observed in the other three subgroups (Table 4).

**TABLE 3:** Associations between TCM decoction use and the stability of within-day GV in the crude analysis, multivariable analysis, and propensity-score analyses.

Analysis	TCM decoctions	Non-TCM decoctions	Or (95% CI)	*P* value
Full cohort				
Unadjusted crude analysis^a^	388	972	3.15 (2.62–3.78)	<0.01
Multivariable analysis^b^	388	972	1.82 (1.66–1.98)	<0.01
Propensity-score analyses				
With matching^c^	278	278	1.77 (1.34–2.33)	<0.01
With inverse probability weighting^d^	327	422	1.63 (1.31–2.04)	<0.01
Adjusted for propensity score^e^	388	972	1.77 (1.58–1.98)	<0.01

^a^ Shown is the odds ratio from the univariate logistic regression, analyzing the association between the use of traditional Chinese medicinal decoctions and the outcomes.

^b^ Shown is the odds ratios from the multivariate logistic regression model, adjusting for 14 variables that were significantly associated with the outcome in univariate analysis (age, BMI, duration of diabetes, systolic blood pressure, HbA1c, FPG, fasting insulin, C-peptide, TCM, syndrome, and comorbidities).

^c^ Shown is the primary analysis with a odds ratios from the multivariate logistic regression model with the same covariates with matching according to the propensity score.

^d^ Shown is the odds ratios from the multivariate logistic regression model with the same covariates with inverse probability weighting according to the propensity score.

^e^ Shown is odds ratios from the multivariate logistic regression model with the same covariates with additional adjustment for the propensity score.

**TABLE 4:** Stability of within-day GV in the propensity-score-matched cohort, according to subgroup.

TCM syndrome	Duration (years)	No. of matched pairs	No. of patients		OR (95% CI)	*P* value
			TCM decoctions	Non-TCM decoctions		
Deficiency syndromes	< 5	70	108	212	2.28 (1.21–4.29)	0.03
Deficiency syndromes	≥5	45	59	346	1.77 (0.80–3.29)	0.23
Phlegm syndromes/liver stagnation and spleen deficiency syndrome/dampness-heat syndrome	<5	80	150	170	1.06 (0.60–1.87)	0.87
Phlegm syndromes/liver stagnation and spleen deficiency syndrome/dampness-heat syndrome	≥5	55	71	243	1.98 (1.08–3,60)	0.06

### Secondary endpoints

In the unadjusted analysis, use of TCM decoctions was associated with a statistically significant reduction in hospital length of stay [odds ratio (OR), 0.93; 95% confidence interval (CI), 0.90 to 0.96; *p* < 0.001; Table 5]. However, this association was not robust, as it was no longer statistically significant after adjusting for confounding variables using propensity score matching (OR, 0.96; 95% CI, 0.91 to 1.01; *p* = 0.22; Table 5) or other methods.

**TABLE 5:** Associations between TCM decoctions use and the length of stay in hospital in the crude analysis, multivariable analysis, and propensity-score analyses.

Analysis	TCM d ecoctions (n)	Non-TCM decoctions (n)	IRR (95% CI)	*P* value
Full cohort				
Unadjusted crude analysis	388	972	0.93 (0.90–0.96)	<0.01
Multivariable analysis	388	972	0.95 (0.92–0.99)	0.01
Propensity-score analyses				
With matching	288	288	0.96 (0.91–1.01)	0.22
With inverse probability weighting	338	443	0.96 (0.94–0.98)	<0.01
Adjusted for propensity score	388	972	0.96 (0.91–1)	0.09

## Discussion

This propensity score-matched cohort study provides valuable insights for clinicians in considering the potential effectiveness of TCM decoctions for maintaining within-day glycemic variability (GV) stability. Our findings suggest an association between TCM decoction use and improved within-day GV stability in hospitalized patients, which persisted after adjusting for potential confounding variables. This association was further corroborated through comprehensive sensitivity analyses. Furthermore, the study revealed that TCM decoction treatment may be most beneficial for patients diagnosed with deficiency syndromes, particularly those with a predominant deficiency of Qi-yin (comprising 74.8% of this subgroup) and a diabetes duration of less than 5 years. Notably, while a small reduction in hospital length of stay was observed among patients receiving TCM decoctions, this effect became statistically non-significant after propensity score matching.

Our study facilitated a more robust investigation into the potential association between TCM decoctions and the stability of within-day GV. The findings hold promise for future clinical practice considerations in T2D management. Strict glycemic control in hospitalized settings, while necessary to minimize hyperglycemia’s detrimental effects in uncontrolled T2D patients (Nusca et al., 2018), can lead to rapid reductions in overall blood glucose levels, potentially increasing within-day GV (Braithwaite, 2013; Akirov et al., 2018). Frequent short-term fluctuations in blood glucose can have a significant impact on various physiological functions. Short-term glucose fluctuations, through the excessive production of reactive oxygen species (ROS), reactive nitrogen species (RNS), inflammatory cytokines, and oxidative stress, can ultimately lead to β-cell apoptosis and a decline in β-cell function (Kohnert et al., 2012), accelerating the deterioration of glycemic control. Additionally, evidence suggests that short-term acute blood glucose fluctuations may induce a greater degree of oxidative stress compared to chronic sustained hyperglycemia, potentially contributing to the development of diabetes complications (Monnier et al., 2006; Ceriello et al., 2008). Individuals with T2D experiencing excessive within-day GV are also at an increased risk of hypoglycemia (Murata et al., 2004; Monnier et al., 2011), lower quality of life and negative moods (Penckofer et al., 2012). Given the significant impact of these adverse effects on T2D management, GV emerges as a crucial target for glycemic control interventions. However, a growing body of research suggests that traditional antidiabetic pharmacotherapies, including basal insulin and intensive treatment strategies, may not necessarily improve GV stability despite achieving glycemic targets (Gerstein et al., 2008; Duckworth et al., 2009; Zenari and Marangoni, 2013). In contrast, our research findings suggest that TCM decoction treatment might offer a unique opportunity to maintain more stable GV, providing a potential approach for future clinical consideration.

The precise mechanisms by which TCM decoctions improve within-day GV stability remain largely unclear. Some studies suggest that TCM may exert regulatory effects that correct internal environment imbalances caused by various pathogenic factors, enabling the body to restore homeostasis more promptly and potentially contribute to reduced glycemic fluctuations (Liu et al., 2015). Our study observed that patients with a shorter disease duration (less than 5 years) and a diagnosis of deficiency syndrome, particularly Qi-Yin deficiency (comprising 74.8% of this subgroup), exhibited greater stability in within-day GV. From the perspective of traditional Chinese medicine (TCM), blood glucose is considered a nutritional substance produced by the transport and transformation function of spleen-stomach (Ke et al., 2022). Therefore, spleen and stomach Qi deficiency is seen as a basic TCM pathogenesis for GV in diabetic patients (Ke et al., 2022). Li, (2023) research on the correlation between symptom scores of Qi deficiency syndrome and blood glucose fluctuations in type 2 diabetes patients revealed a positive correlation between Qi deficiency and within-day GV. Similarly, Zhang et al. (2024) found that patients with severe Yin deficiency exhibited greater glycemic fluctuations. Zheng, (2023) study further corroborated these findings, showing that patients with deficiency syndromes, particularly those with both Qi and Yin deficiencies, experienced significant within-day GV. Additionally, a clinical study by Pang et al. indicated that patients with Qi and Yin deficiency who received TCM treatment aimed at replenishing Qi and nourishing Yin exhibited the least within-day GV (Pang and Sun, 2017). These findings collectively suggest a significant positive correlation between the severity of Qi and Yin deficiencies and the stability of glycemic levels. Correcting the states of Qi and Yin deficiencies through TCM treatment could therefore be effective in maintaining stable glycemic levels in type 2 diabetes patients. Furthermore, patients with a shorter disease duration, whose islet function is not yet severely compromised, tend to have relatively stable glycemic levels, and may experience more pronounced benefits from TCM treatments (Zhang et al., 2015). However, limited clinical research currently exists regarding the efficacy of TCM in specifically reducing glycemic fluctuations, and the underlying mechanisms are yet to be fully elucidated. Additionally, no prior studies have definitively identified superior glycemic stability with TCM interventions in specific patient populations.

Our analysis revealed a small reduction in the length of hospital stay for patients receiving TCM decoctions. However, this finding did not reach statistical significance. Furthermore, propensity score matching, which aimed to account for potential confounding variables, resulted in the loss of this observed association. Therefore, our results should be interpreted with caution and further research is warranted to validate this potential benefit of TCM decoctions on hospital stay duration.

This study possesses several key strengths. First, we leveraged a population-based cohort exclusively receiving TCM decoctions. This unique design allowed for a direct comparison of outcomes between patients receiving TCM decoctions and those receiving concurrent antidiabetic Western medications (controls) using a rigorous propensity score matching approach. Furthermore, we employed a series of analyses utilizing various propensity score methods to enhance the robustness and reliability of our findings. The consistency observed across these multiple sensitivity analyses strengthens our confidence in the results.

Our study has several limitations that should be acknowledged. First, the lack of access to a larger population dataset restricted our ability to subdivide the control group into categories based on specific Western medications. This limited our capacity to directly compare the outcomes of traditional Chinese medicine decoctions with a particular anti-diabetic medication. Second, the absence of continuous glucose monitoring (CGM) data is a noteworthy limitation. CGM data provides minute-by-minute glycemic fluctuations, offering a more precise metric for assessing glycemic variability compared to the SMBG employed in this study. Additionally, limitations include missing data for certain variables and the potential for inaccuracies in electronic health records. We have addressed missing data to minimize bias, but this remains a potential source of error. Finally, the single-center observational design may limit the generalizability of our results. Future studies with larger cohorts, extended follow-up periods, and the incorporation of CGM data are necessary to further elucidate the relationship between TCM decoction therapy and enhanced GV stability. These studies would strengthen the evidence for the potential of TCM interventions to achieve better glycemic maintenance and stability in hospitalized type 2 diabetes patients. Additionally, further research is needed to understand the mechanisms underlying these potential associations.

In conclusion, our study demonstrates that, among hospitalized patients diagnosed with type 2 diabetes, TCM decoctions are more efficacious in maintaining stable within-day GV compared to antidiabetic medications derived from Western medicine. This beneficial effect is particularly pronounced in patients presenting with deficiency syndrome and a disease course of less than 5 years. However, it is important to note that our findings do not support a reduction in hospitalization duration associated with TCM decoction therapy.

## Data Availability

The raw data supporting the conclusions of this article will be made available by the authors, without undue reservation.
