# Supplementary material for: Observational study on stability of within-day glycemic variability of type 2 diabetes inpatients treated with decoctions of traditional Chinese medicine
Source: Front Pharmacol. 2024 Jul 19;15:1378140. doi: 10.3389/fphar.2024.1378140 (PMC11294233; doi:10.3389/fphar.2024.1378140)
Supplement: Supplementary file 1 [file Table1.docx]

| Table S1 Composition of decoctions. | |
| --- | --- |
| Decoction Name | Composition |
| Qingre Yangyin Tiaotang Decoction | 30 g of Gypsum Fibrosum, 10 g of Anemarrhena asphodeloides Bunge [Asparagaceae; Anemarrhenae rhizoma], 10 g of Rehmannia glutinosa (Gaertn.) DC. [Orobanchaceae; Rehmanniae Radix Praeparata], 30 g of Ophiopogon japonicus (Thunb.) Ker Gawl. [Asparagaceae; Ophiopogonis Radix], 30 g of Achyranthes bidentata Blume [Amaranthaceae; Achyranthis Bidentatae Radix], 30 g of Pseudostellaria heterophylla (Miq.) Pax [Caryophyllaceae; Pseudostellariae radix], 20 g of Pueraria montana var. lobata (Willd.) Maack & Sanjappa [Fabaceae; Puerariae radix], 15 g of Trichosanthes kirilowii Maxim. [Cucurbitaceae; Trichosanthis radix], 10 g of Atractylodes lancea (Thunb.) DC. [Asteraceae; Atractylodis Rhizoma], 10 g of Citrus aurantium L. [Rutaceae; Aurantii fructus], 3 g of Cimicifuga heracleifolia Kom. [Ranunculaceae; Cimicifugae Rhizoma], 6 g of Glycyrrhiza glabra L. [Fabaceae; Glycyrrhizae radix et rhizoma] |
| Yiqi Yangyin Tiaotang Decoction | 30 g of Pseudostellaria heterophylla (Miq.) Pax [Caryophyllaceae; Pseudostellariae radix], 30 g of Astragalus membranaceus (Fisch.) Bunge [Fabaceae; Astragali radix], 30 g of Rehmannia glutinosa (Gaertn.) DC. [Orobanchaceae; Rehmanniae Radix Praeparata], 30 g of Cornus officinalis Siebold & Zucc. [Cornaceae; Corni Fructus], 20 g of Dioscorea oppositifolia L. [Dioscoreaceae; Dioscoreae Rhizoma], 15 g of Atractylodes lancea (Thunb.) DC. [Asteraceae; Atractylodis Rhizoma], 30 g of Atractylodes macrocephala Koidz. [Asteraceae; Atractylodis Macrocephalae Rhizoma], 10 g of Alisma plantago-aquatica [Alismataceae; Alismatis rhizoma], 30 g of Salvia miltiorrhiza Bunge [Lamiaceae; Salviae Miltiorrhizae Radix et Rhizoma], 50 g of Wolfiporia cocos (Schwein.) Ryvarden & Gilb. [Polyporaceae; Poria], 10 g of Citrus aurantium L. [Rutaceae; Aurantii fructus], 10 g of Ophiopogon japonicus (Thunb.) Ker Gawl. [Asparagaceae; Ophiopogonis Radix], 6 g of Cimicifuga heracleifolia Kom. [Ranunculaceae; Cimicifugae Rhizoma] |
| Shugan Jianpi Tiaotang Decoction | 10 g of Bupleurum chinense DC. [Apiaceae; Bupleuri Radix], 10 g of Angelica sinensis (Oliv.) Diels [Apiaceae; Angelicae sinensis radix], 15 g of Wolfiporia cocos (Schwein.) Ryvarden & Gilb. [Polyporaceae; Poria], 10 g of Paeonia lactiflora Pall. [Paeoniaceae; Paeoniae Radix Alba], 3 g of Atractylodes lancea (Thunb.) DC. [Asteraceae; Atractylodis Rhizoma], 10 g of Atractylodes macrocephala Koidz. [Asteraceae; Atractylodis Macrocephalae Rhizoma], 10 g of Paeonia suffruticosa Andr. [Paeoniaceae; Moutan Cortex], 6 g of Gardenia jasminoides J. Ellis [Rubiaceae; Gardeniae fructus], 10 g of Glycine max (L.) Merr. [Fabaceae; Sojae Semen Preparatum], 30 g of Achyranthes bidentata Blume [Amaranthaceae; Achyranthis Bidentatae Radix], 6 g of Mentha haplocalyx Briq. [Lamiaceae; Menthae Herba], 6 g of Glycyrrhiza glabra L. [Fabaceae; Glycyrrhizae radix et rhizoma], 6 g of Cimicifuga heracleifolia Kom. [Ranunculaceae; Cimicifugae Rhizoma], 10 g of Zingiber officinale Roscoe [Zingiberaceae; Zingiberis Rhizoma Recens (fresh ginger root)] |
| Hezhong Jiangzhuo Tiaotang Decoction | 30 g of Atractylodes lancea (Thunb.) DC. [Asteraceae; Atractylodis Rhizoma], 30 g of Atractylodes macrocephala Koidz. [Asteraceae; Atractylodis Macrocephalae Rhizoma], 10 g of Citrus reticulata Blanco [Rutaceae; Citri Reticulatae Pericarpium], 10 g of Magnolia officinalis Rehder & E.H. Wilson [Magnoliaceae; Magnoliae officinalis cortex], 30 g of Alisma plantago-aquatica [Alismataceae; Alismatis rhizoma], 30 g of Polyporus umbellatus (Pers.) Fr. [Polyporaceae; Polyporus], 30 g of Wolfiporia cocos (Schwein.) Ryvarden & Gilb. [Polyporaceae; Poria], 6 g of Cinnamomum cassia (L.) J. Presl [Lauraceae; Cinnamomi ramulus], 30 g of Coix lacryma-jobi L. var. ma-yuen [Poaceae; Coicis semen], 10 g of Pinellia ternata (Thunb.) Makino [Araceae; Pinelliae Rhizoma], 10 g of Gleditsia sinensis Lam. [Fabaceae; Gleditsiae spina], 30 g of Achyranthes bidentata Blume [Amaranthaceae; Achyranthis Bidentatae Radix], 3 g of Cimicifuga heracleifolia Kom. [Ranunculaceae; Cimicifugae Rhizoma], 3 g of Glycyrrhiza glabra L. [Fabaceae; Glycyrrhizae radix et rhizoma] |
| Qingre Huashi Tiaotang Decoction | 15 g of Coptis chinensis Franch. [Ranunculaceae; Coptidis rhizoma], 10 g of Magnolia officinalis Rehder & E.H. Wilson [Magnoliaceae; Magnoliae officinalis cortex], 10 g of Gardenia jasminoides J. Ellis [Rubiaceae; Gardeniae fructus], 10 g of Pinellia ternata (Thunb.) Makino [Araceae; Pinelliae Rhizoma], 30 g of Coix lacryma-jobi L. var. ma-yuen [Poaceae; Coicis semen], 10 g of Phellodendron amurense Rupr. [Rutaceae; Phellodendri amurensis cortex], 10 g of Atractylodes lancea (Thunb.) DC. [Asteraceae; Atractylodis Rhizoma], 10 g of Citrus aurantium L. [Rutaceae; Aurantii fructus immaturus], 6 g of Acorus gramineus aiton [Acoraceae; Acori calami rhizoma], 30 g of Phragmites australis (Cav.) Trin. ex Steud. [Poaceae; Phragmitis Rhizoma], 30 g of Achyranthes bidentata Blume [Amaranthaceae; Achyranthis Bidentatae Radix], 6 g of Cimicifuga heracleifolia Kom. [Ranunculaceae; Cimicifugae Rhizoma] |
| Jianpi Yishen Tiaotang Decoction | 30 g of Pseudostellaria heterophylla (Miq.) Pax [Caryophyllaceae; Pseudostellariae radix], 60 g of Astragalus membranaceus (Fisch.) Bunge [Fabaceae; Astragali radix], 30 g of Dioscorea oppositifolia L. [Dioscoreaceae; Dioscoreae Rhizoma], 30 g of Rehmannia glutinosa (Gaertn.) Libosch. [Orobanchaceae; Rehmanniae radix praeparata], 30 g of Cornus officinalis Siebold & Zucc. [Cornaceae; Corni Fructus], 30 g of Alisma plantago-aquatica [Alismataceae; Alismatis rhizoma], 30 g of Achyranthes bidentata Blume [Amaranthaceae; Achyranthis Bidentatae Radix], 30 g of Atractylodes lancea (Thunb.) DC. [Asteraceae; Atractylodis Rhizoma], 30 g of Atractylodes macrocephala Koidz. [Asteraceae; Atractylodis Macrocephalae Rhizoma], 10 g of Citrus aurantium L. [Rutaceae; Aurantii fructus], 30 g of Polyporus umbellatus (Pers.) Fr. [Polyporaceae; Polyporus], 30 g of Wolfiporia cocos (Schwein.) Ryvarden & Gilb. [Polyporaceae; Poria], 30 g of Mantidis Oötheca, 3 g of Cimicifuga heracleifolia Kom. [Ranunculaceae; Cimicifugae Rhizoma] |
| Shenqi Dihuang Decoction | 30 g of Codonopsis pilosula (Franch.) Nannf. [Campanulaceae; Codonopsis Radix], 30 g of Astragalus membranaceus (Fisch.) Bunge [Fabaceae; Astragali radix], 30 g of Wolfiporia cocos (Schwein.) Ryvarden & Gilb. [Polyporaceae; Poria], 30 g of Rehmannia glutinosa (Gaertn.) DC. [Orobanchaceae; Rehmanniae Radix Praeparata], 15 g of Dioscorea oppositifolia L. [Dioscoreaceae; Dioscoreae Rhizoma], 10 g of Paeonia suffruticosa Andr. [Paeoniaceae; Moutan Cortex], 30 g of Cornus officinalis Siebold & Zucc. [Cornaceae; Corni Fructus], 10 g of Alisma plantago-aquatica [Alismataceae; Alismatis rhizoma] |
| Shengmaiyin | 30 g of Codonopsis pilosula (Franch.) Nannf. [Campanulaceae; Codonopsis Radix], 30 g of Ophiopogon japonicus (Thunb.) Ker Gawl. [Asparagaceae; Ophiopogonis Radix], 30 g of Schisandra chinensis (Turcz.) Baill. [Schisandraceae; Schisandrae fructus] |
| Buyang Huanwu Decoction | 30 g of Astragalus membranaceus (Fisch.) Bunge [Fabaceae; Astragali radix], 15 g of Paeonia lactiflora Pall. [Paeoniaceae; Paeoniae Radix Rubra], 12 g of Ligusticum chuanxiong Hort. [Apiaceae; Chuanxiong Rhizoma], 15 g of Angelica sinensis (Oliv.) Diels [Apiaceae; Angelicae sinensis radix], 8 g of Pheretima, 10 g of Prunus persica (L.) Batsch [Rosaceae; Persicae semen], 10 g of Carthamus tinctorius L. [Asteraceae; Carthami flos] |
| Gualou Xiebai Banxia Decoction | 30 g of Trichosanthes kirilowii Maxim. [Cucurbitaceae; Trichosanthis fructus], 12 g of Allium macrostemon Bunge [Amaryllidaceae; Allii macrostemonis bulbus], 10 g of Pinellia ternata (Thunb.) Makino [Araceae; Pinelliae Rhizoma] |
| Taohong Siwu Decoction | 15 g of Angelica sinensis (Oliv.) Diels [Apiaceae; Angelicae sinensis radix], 10 g of Rehmannia glutinosa (Gaertn.) DC. [Orobanchaceae; Rehmanniae Radix Praeparata], 10 g of Ligusticum chuanxiong Hort. [Apiaceae; Chuanxiong Rhizoma], 30 g of Paeonia lactiflora Pall. [Paeoniaceae; Paeoniae Radix Alba], 15 g of Prunus persica (L.) Batsch [Rosaceae; Persicae semen], 10 g of Carthamus tinctorius L. [Asteraceae; Carthami flos] |
| Qiju Dihuang Decoction | 15 g of Lycium barbarum L. [Solanaceae; Lycii Fructus], 15 g of Chrysanthemum morifolium Ramat. [Asteraceae; Chrysanthemi Flos], 15 g of Wolfiporia cocos (Schwein.) Ryvarden & Gilb. [Polyporaceae; Poria], 30 g of Rehmannia glutinosa (Gaertn.) DC. [Orobanchaceae; Rehmanniae Radix Praeparata], 30 g of Dioscorea oppositifolia L. [Dioscoreaceae; Dioscoreae Rhizoma], 10 g of Paeonia suffruticosa Andr. [Paeoniaceae; Moutan Cortex], 30 g of Cornus officinalis Siebold & Zucc. [Cornaceae; Corni Fructus], 15 g of Alisma plantago-aquatica [Alismataceae; Alismatis rhizoma] |
| Linggui Zhugan Decoction | 20 g of Wolfiporia cocos (Schwein.) Ryvarden & Gilb. [Polyporaceae; Poria], 20 g of Atractylodes macrocephala Koidz. [Asteraceae; Atractylodis Macrocephalae Rhizoma], 20 g of Cinnamomum cassia (L.) J. Presl [Lauraceae; Cinnamomi ramulus], 10 g of Glycyrrhiza glabra L. [Fabaceae; Glycyrrhizae radix et rhizoma] |
| Danggui Sini Decoction | 15 g of Angelica sinensis (Oliv.) Diels [Apiaceae; Angelicae sinensis radix], 12 g of Cinnamomum cassia (L.) J. Presl [Lauraceae; Cinnamomi ramulus], 45 g of Paeonia lactiflora Pall. [Paeoniaceae; Paeoniae Radix Alba], 5 g of Asarum heterotropoides F.Schmidt [Aristolochiaceae; Asari Radix et Rhizoma], 6 g of Tetrapanax papyrifer (Hook.) K.Koch [Araliaceae; Medulla Tetrapanacis], 10 g of Glycyrrhiza glabra L. [Fabaceae; Glycyrrhizae radix et rhizoma] |
| Siwu Decoction | 10 g of Angelica sinensis (Oliv.) Diels [Apiaceae; Angelicae sinensis radix], 10 g of Paeonia lactiflora Pall. [Paeoniaceae; Paeoniae Radix Alba], 6 g of Ligusticum chuanxiong Hort. [Apiaceae; Chuanxiong Rhizoma], 10 g of Rehmannia glutinosa (Gaertn.) Libosch. [Orobanchaceae; Rehmanniae radix praeparata] |

| Table S2 Univariate analysis of primary and secondary outcomes. | | |
| --- | --- | --- |
| Characteristic | primary outcome | secondary outcome |
|  | P value | P value |
| **Age** | <0.01 | 0.87 |
| **Sex** |  |  |
| Female | NA | NA |
| Male | 0.38 | 0.65 |
| **Body-mass index** -- kg/m^2^ |  |  |
| ≤18 and 18-23.9 | NA | NA |
| 24-27.9 | <0.01 | 0.28 |
| ≥28 | <0.01 | 0.19 |
| **Duration of diabetes** -- year |  |  |
| ＜3 | NA | NA |
| 3-5 | 0.06 | 0.09 |
| 5-10 | <0.01 | 0.01 |
| ≥10 | <0.01 | 0.66 |
| **Systolic blood pressure** -- mm HG | 0.02 | 0.20 |
| **HbA1c** -- % | <0.01 | <0.01 |
| **FPG** -- mmol/L | <0.01 | 0.03 |
| **Fasting insulin** -- μIU/ml |  |  |
| ＜10 | NA | NA |
| 10-15 | 0.01 | 0.48 |
| ＞15 | <0.01 | 0.22 |
| **LDL** -- mmol/L |  |  |
| ＜2.6 | NA | NA |
| ≥2.6 | 0.73 | <0.01 |
| **C-peptide** -- ng/mL |  |  |
| ＜1.17 | 0.29 | 0.02 |
| 1.17-2.51 | NA | NA |
| ≥2.51 | <0.01 | 0.55 |
| **TCM syndrome** |  |  |
| deficiency syndromes | NA | NA |
| phlegm syndromes | <0.01 | 0.76 |
| liver stagnation and spleen deficiency syndrome | 0.01 | 0.07 |
| dampness-heat syndrome | <0.01 | 0.43 |
| **Comorbidities** |  |  |
| Liver Diseases | 0.02 | 0.61 |
| Hypertension | 0.3 | 0.84 |
| Hyperlipidemia | 0.04 | 0.96 |
| osteoarthropathia | 0.98 | 0.63 |
| Coronary Atherosclerosis | 0.01 | 0.60 |
| Chronic Kidney Disease | <0.01 | <0.01 |
| Cerebral Infarction | 0.01 | 0.51 |
| Ischemic cerebrovascular disease | 0.07 | <0.01 |
| Ischemic heart disease | 0.79 | 0.52 |
| Diabetic retinopathy | <0.01 | 0.12 |
| Diabetic macrovascular disease | 0.26 | 0.24 |
| Diabetic polyneuropathy | 0.34 | 0.77 |
| Diabetic peripheral vascular disease | 0.86 | 0.37 |

| Table S3 Sensitivity analyses of the associations between TCM Decoctions Use and the stability of within-day GV. | | | | | | |
| --- | --- | --- | --- | --- | --- | --- |
|  |  | Analysis | TCM  Decoctions | Non-TCM Decoctions | OR (95%CI) | *P* value |
| **Additional outcome** | PPGE | **Full cohort** |  |  |  |  |
|  |  | Unjusted crude analysis | 388 | 972 | 2.32 (1.93-2.8) | < 0.01 |
|  |  | Multivariable analysis | 388 | 972 | 1.77 (1.62-1.94) | < 0.01 |
|  |  | **Propensity-score analyses** |  |  |  |  |
|  |  | With matching | 277 | 277 | 1.70  (1.29-2.24) | < 0.01 |
|  |  | With inverse probability weighting | 324 | 422 | 1.61 (1.29-2.02) | < 0.01 |
|  |  | Adjusted for propensity score | 388 | 972 | 1.69 (1.51-1.9) | < 0.01 |
|  | LAGE | **Full cohort** |  |  |  |  |
|  |  | Unjusted crude analysis | 388 | 972 | 4.19 (3.48-5.06) | < 0.01 |
|  |  | Multivariable analysis | 388 | 972 | 1.87 (1.71-2.05) | < 0.01 |
|  |  | **Propensity-score analyses** |  |  |  |  |
|  |  | With matching | 280 | 280 | 1.94 (1.52-2.48) | < 0.01 |
|  |  | With inverse probability weighting | 325 | 415 | 1.96 (1.75-2.2) | < 0.01 |
|  |  | Adjusted for propensity score | 388 | 972 | 1.77 (1.42-2.20) | < 0.01 |
|  | | | | | | |
| **Complete dataset** |  | **Full cohort** |  |  |  |  |
|  |  | Unjusted crude analysis | 328 | 737 | 3.07 (2.41-3.91) | < 0.01 |
|  |  | Multivariable analysis | 328 | 737 | 1.81 (1.37-2.40) | < 0.01 |
|  |  | **Propensity-score analyses** |  |  |  |  |
|  |  | With matching | 233 | 233 | 1.69 (1.27-2.25) | < 0.01 |
|  |  | With inverse probability weighting | 443 | 592 | 1.71 (1.37-2.14) | < 0.01 |
|  |  | Adjusted for propensity score | 328 | 737 | 1.78 (1.28-2.47) | < 0.01 |
